# Medical cannabis in Israel: a comprehensive review of trends and regulations, 2011–2025

**DOI:** 10.1186/s42238-025-00344-1

**Published:** 2025-11-13

**Authors:** Joshua Aviram

**Affiliations:** Department of Nursing, Faculty of Health Sciences, Laboratory of Medical Cannabis, Pain and Sleep Relationships Research, Ariel University, Ariel, 40700 Israel

**Keywords:** Medical cannabis, Israel, Regulatory reform, Prescribing patterns, Chronic non-cancer pain, Post-traumatic stress disorder, Dosage trends, Cannabis policy

## Abstract

**Background:**

Since its initial approval in 1992, medical cannabis (MC) in Israel has undergone extensive regulatory and structural transformation. This study aims to present the most comprehensive retrospective analysis to date of the evolving MC policy framework in Israel and its impact on patient access, prescribing patterns, and treatment characteristics from 2011 to 2025.

**Methods:**

The study included a longitudinal review and secondary analysis of monthly Israeli Medical Cannabis Unit (IMCU) reports, covering key parameters such as the number of active licenses, indications, dosage patterns, product types, healthcare provider involvement, and importation data. Regulatory milestones and reforms were mapped to observed shifts in these parameters, with particular attention to the transition to per-product pricing (2019), the decentralization of prescription authority to trained physicians (2020), and the Health Maintenance Organization (HMO)-led prescribing reform (2024).

**Results:**

The number of active MC licenses increased markedly by over 4,400% from 3,097 in 2011 to a peak of 140,483 in January 2024. This growth coincided with expanded indications, broader pharmacy access, and prescribing authority beyond IMCU physicians. The 2024 HMO-led reform, followed by a 7.5% gradual decline to 129,900 active MC licenses and prescriptions on March 2025, alongside incomplete reporting of prescription indications data. This decline is consistent with this study hypothesis of stricter HMO gatekeeping and transition frictions. The most frequently approved indication was chronic non-cancer pain (CNCP) (increasing from 53 to 63%), with substantial, constant growth of ~ 89% (from 9 to 17%) in the second most common indication, post-traumatic stress-disorder. Approved monthly dosages shifted significantly: the share of patients receiving 50gr/month rose by ~ 108% and 60gr/month by ~ 117%, while the 40gr/month category consistently comprised ~ 22–25% of active patient licenses. Product form trends were also notable: flower-based MC products accounted for over 94% of usage by 2025. At its peak, the estimated annual market value of MC products ranged between $252–$684 million.

**Conclusions:**

Israel’s MC program reflects a dynamic interplay between policy reforms and patient treatment patterns. While preliminary MC medicalization improved access, recent decentralization efforts have introduced new challenges related to oversight and data transparency, plausibly reflecting stricter HMO gatekeeping, transition frictions, and the post-investigation non-renewal of irregular licenses (hypothesis given current data gaps), that may be associated with a decline in active MC licenses. The findings underscore the importance of maintaining consistent national reporting standards to guide evidence-based MC policy and clinical practice.

## Introduction

Botanical medical cannabis (MC) regulation and medicalization have been promoted in the past decades in the United States (US) (Boehnke et al. 2022) and across Europe (Farber 2024). In this paper, “botanical” MC refers to whole-plant herbal cannabis products (dried flower and oil/extracts derived from the plant flowers) and excludes synthetic cannabinoids and regulatory approved cannabis-based medicines (i.e., Nabiximols, Epidiolex, Dronabinol, and Nabilone). Despite this process, MC is categorized as a schedule I drug, meaning, as an illicit substance with no medicinal value (DEA 2018). As such, many countries, alongside the approval of MC for medical purposes, for various reasons, also constructed registries, with publicly available information to monitor certain aspects of the treatment (Boehnke et al. 2025).

In Israel, MC has been regulated and approved for medical purposes since 1992, under specific conditions (Zolotov et al 2021). A previous paper on the trends in MC licensure between the years 2013–2018, based on publicly available data sourced by the movement for freedom of information (Freedom of information movement 2018) for the annual changes of active MC licenses, gender, age and medical indications presented an increase of 121% in MC licenses, decrease in the frequencies in MC licenses for patients aged 41–65 and an increase in frequency for patients aged over 66 years, dominance of male (68.5%) patients and an increase of MC licenses frequencies for chronic non-cancer pain (CNCP) and post-traumatic stress disorder (PTSD), while undefined (i.e., "other") medical indication category decreased in frequency (Sznitman 2020).

Since the establishment of the Israeli MC regulations, endorsing research, many basic science (Milay et al. 2020; Berman et al. 2018; Shapira 2019; Raz 2023), real world evidence, observational trials, many of those published by the author of the current study (Aviram et al. 2022a; Aviram et al. 2020a; Aviram et al. 2022b; Aviram et al. 2023; Aviram 2021 Bar-Lev Schleider et al. 2022; Sagy et al. 2019; Haroutounian et al. 2016; Pud et al 2024) and clinical trials (Hermush et al. 2022; Naftali et al. 2021; Aran et al 2021; Eisenberg et al 2014) has been published, with most clinical results demonstrating general improvement in the wellbeing of patients. For botanical MC, observational studies vastly outnumber randomized controlled trials (RCTs), reflecting legal (National Academies of Sciences Engineering and Medicine 2017), economic, and technical barriers to large scale, high-quality RCTs (Piomelli et al. 2019; Fortin et al 2020). This asymmetry helps explain gaps between RCTs findings and real-world observational results. Notably, most observational trials cited above reported reduction in pain intensity of chronic pain and cancer pain patients, improvement in quality of life, decrease in sleep disturbance and improvement in many other facets of health. Nonetheless, one of most recent and most inclusive meta-analyses (including 65 studies with 7,017 patients total), examined the efficacy and safety of cannabinoids via results of RCTs and concluded that "The harmful effects of cannabinoids for pain seem to outweigh the potential benefits" (Barakji et al. 2023). Importantly, there is a gap between those perspectives as observational studies are mostly conducted with botanical MC, whereas RCTs data is mostly based on cannabis derivates and synthetic cannabinoids, which has a different efficacy and safety profile (Aviram and Samuelly-Leichtag 2017). This controversy is one of the main conflicts points between the growing numbers in patients being treated with MC and its regulatory status as un-authorized medication. Additionally, there is a market failure in clinical research on botanical cannabis which may persist for decades because the above legal, economic and technical barriers which depress incentives for high-quality RCTs (National Academies of Sciences Engineering and Medicine 2017; Piomelli et al. 2019; Fortin and Massim 2020).

The status of MC and many aspects of it, including regulation by the Israeli ministry of health (IMOH) and by the Israeli medical cannabis unit (IMCU), oversight of ministry of public security, Israeli drug enforcement administration, the police and the involvement of Knesset members, physician organizations, patients, medical practitioners and MC growers/companies is a permanent subject of debate and changes (Zarhin et al. 2017).

A central factor influencing MC licensure trends is likely the degree of adoption within the medical community. From a survey that was published on 2015, Israeli physicians with a range of specialties reported that most (~ 90%) came across at least one MC patient and that more than half (~ 59%) recommended to the IMCU for a MC license for at least one patient. Study analysis showed that the only factor that positively affected physicians on their decision on whether to recommend MC was past experience with patients, meaning that physicians that were positive about prescribing MC are more likely to stay positive. Generally, over 75% agreed on the therapeutic potential of MC and that it’s a legitimate medical treatment (Ebert et al. 2015). Hence, it could be expected that MC will be generally endorsed by physicians in Israel in the following years.

Given that nearly a decade has passed since the last publication analyzing licensure trends, and new monthly (rather than annual) data are now available, the objective of the current study is to present the temporal trends of all relevant available information and to investigate the relationships between these trends and regulatory changes.

## Methods

Since December 2020 through June 2025, the IMCU has published monthly reports (PDF) on their official website that contain information on specific characteristics of patients holding valid Israeli MC licenses (Israeli Ministry Of Health 2025). As of September 15, 2025, data for certain months were missing for the above-mentioned study scope. Several of these missing reports were recovered through internet searches from external repositories (Israeli Ministry Of Health 2020; Israeli Ministry Of Health 2021a; Israeli Ministry Of Health 2021b; Israeli Ministry Of Health et al. 2021c). The reports that are missing (due to being either unpublished or not disseminated) included: January, May and July of 2021; and February, April, July and August of 2025. Notably, as post-Health Maintenance Organizations (HMO) reform report of June 2025 did not provide total licenses number fractionation between HMOs and IMCU (Israeli Ministry Of Health 2025), it could not be analyzed.

The available information from the reports is detailed below.

### Trends in MC licensure and active prescriptions

The trends and numbers of patients with a valid/active MC license have been reported for the specific month the report was issued, alongside a varying amount of retrospective data, differing from one report to the next. For example, the earliest report from December of 2020, provided information going back to April 2011. For these reports, some months data was not available and could not be extrapolated from the figure. When a formal report was available, the number was extracted from it and not from the retrospective data of later reports. Any relevant regulatory change was presented with this information. Notably, following April 2024, a reform reallocated most medical indications to the authority of the Israeli HMOs for a prescription model (Israeli government 2024), except for CNCP and PTSD, which remained under the IMCU authority. Although the rationale for this decision has not been explained, it might be assumed to reflect plausible higher prevalence burden that the HMOs were not yet ready to assume responsibility on. Only in the reports from December 2024, and January and March 2025, were the numbers of patients with a valid prescription reported, but it could not be extrapolated for the interim period back to the initiation of this reform.

### Gender of patients with active MC licenses

Gender numeric information was available in the reports between August 2024 to March 2025. It is displayed in percentage of the total active MC licenses to allow temporal comparison.

### Medical indications of active MC licenses

Medical indications information was available in the monthly reports between December 2020 and March 2025. Indications include CNCP, "unknown/undefined" indication category, oncology disease symptoms, PTSD, oncology chemotherapy adverse events, Crohn's disease, Parkinson's disease, multiple sclerosis (MS), palliative treatment, Tourette syndrome, adult epilepsy, colitis, children epilepsy, human immunodeficiency virus (HIV) cachexia, glaucoma, autistic spectrum disorder (ASD) and dementia. It is displayed in percentage of the total active MC licenses to allow temporal comparison.

### MC monthly allowance/quantities categories

In the monthly reports, a figure was reported on the distribution of approved monthly allowance (allowance of weight range in grams) per patient. Quantities were reported in the categories: 1–10, 11–20, 21–30, 31–40, 41–50, 51–60, 61–70, 71–80, 81–90, 91–100, 101–110 and over 110 g. These values correspond with 10, 20, 30, 40, 50, 60, 70, 80, 90, 100, 110 or over 110 g (gr), respectively, of MC products that patients are allowed to buy during each month. It is displayed in percentage of the total active MC licenses to allow temporal comparison.

### HMOs of patients with active MC licenses

Information on the HMOs was available in the monthly reports between December 2020 and March 2025. The categories in these reports are for Clalit, Maccabi, Meuhedet, Leumit and "other". As there is no additional HMO in Israel, "other" likely refers to missing or unclassified data. It is displayed in percentage of the total active MC licenses to allow temporal comparison.

Although the HMOs MC prescription reform was applied in April 2024 (Israeli government 2024), the number of MC prescriptions per HMO was reported only in the reports from December 2024, and January and March 2025. Based on this data, a calculation was performed combining these figures with the number of MC licenses to provide a clearer display of the status.

### Cumulative MC quantity allowance based on administration routes of active MC licenses

Since its inception, the IMCU permitted the use of specific MC products. Specifically, dried flowers to be utilized by smoking or vaporizing, oil extracts and at a certain period, cookies for children (Landshaft et al 2019). A metered-dose inhaler was also approved in 2019 (Aviram et al. 2022b). In addition, the authorizing physician approves the allowed MC quantity per month for purchase of each product type that is permitted in the MC license.

Information on the cumulative MC quantity allowance (Kilograms, Kgs) based on administration routes of active MC licenses was reported alongside a varying amount of retrospective data. As there was no way to assess the most accurate reported numbers, the information was extracted for consecutive periods which provided information from August 2019 up to March 2025. Information was provided for total monthly allowance of all product types, and by MC extract/oil, MC flowers, and MC inhaler categories. Percentage is calculated out of the total quantity allowance, per product type.

MC in Israel is sold in units of the 10gr per package for flowers, bottles of oil/extracts are sold in units of 10 ml (ml), this is regulatory equivalent to 10gr for the purpose of this report.

Inhaler category was not indicated in numerically in the reports, but as this was the only category not being reported, it was calculated.

In the reports between August 2019 and October 2019, data on types of products available by active licenses separated oil and extract. As these are essentially the same product type, they were merged. This is justified by the IMCU official correspondence as an error in the reports. Between September 2021 and March 2025, only "extract" was reported, and was similarly merged with the oil category.

For flowers and oil combined, based on the above assumptions, a calculation was performed to extrapolate the amount of product types available by the MC license approval each month per patient.

### Percentage of sales based on quantity allowance of MC for active MC licenses

Information on the proportion of sales relative to licensed MC amounts was available in the reports between August 2024 and March 2025. It was reported retrospectively but whenever available, extracted from the specific monthly reports. As this information is limited to a few time points, it was not used to adjust estimates of the number of product types sold each month per patient.

### MC import to Israel

Notably, the only MC product type being imported to Israel is MC flowers (Israeli government 2024). MC flowers import information was available in the reports between January 2024 and March 2025. Information is reported in grams of product. Based on the data that one unit of 10gr equals one package, the number of packages imported was calculated. It was also converted into Kgs for comparability with domestic data.

Based on calculations performed, this information is in addition to the report for cumulative MC quantity allowance based on administration routes of active MC licenses and not part of the total Kgs based on active MC licenses. As it is not clear if those amounts were purchased by patients, or what percentage of it, they were not included in the calculation of the number of products sold each month per patient.

### MC regulatory assessments

MC regulatory developments in Israel were assessed based on information extracted from official government, IMOH, and IMCU websites. When official sources were unavailable, supplementary information was obtained and cited from external repositories and credible press publications.

### Statistical analysis

R software (V.1.1.463) with atable (Ströbel et al 2019), ggplot (Patil et al 2008) and tidyverse (Wickham et al 2019) packages were used for analyses. Only epidemiological and descriptive data is displayed.

## Results

### Trends in MC licensure and active prescriptions

**Fig. 1:**
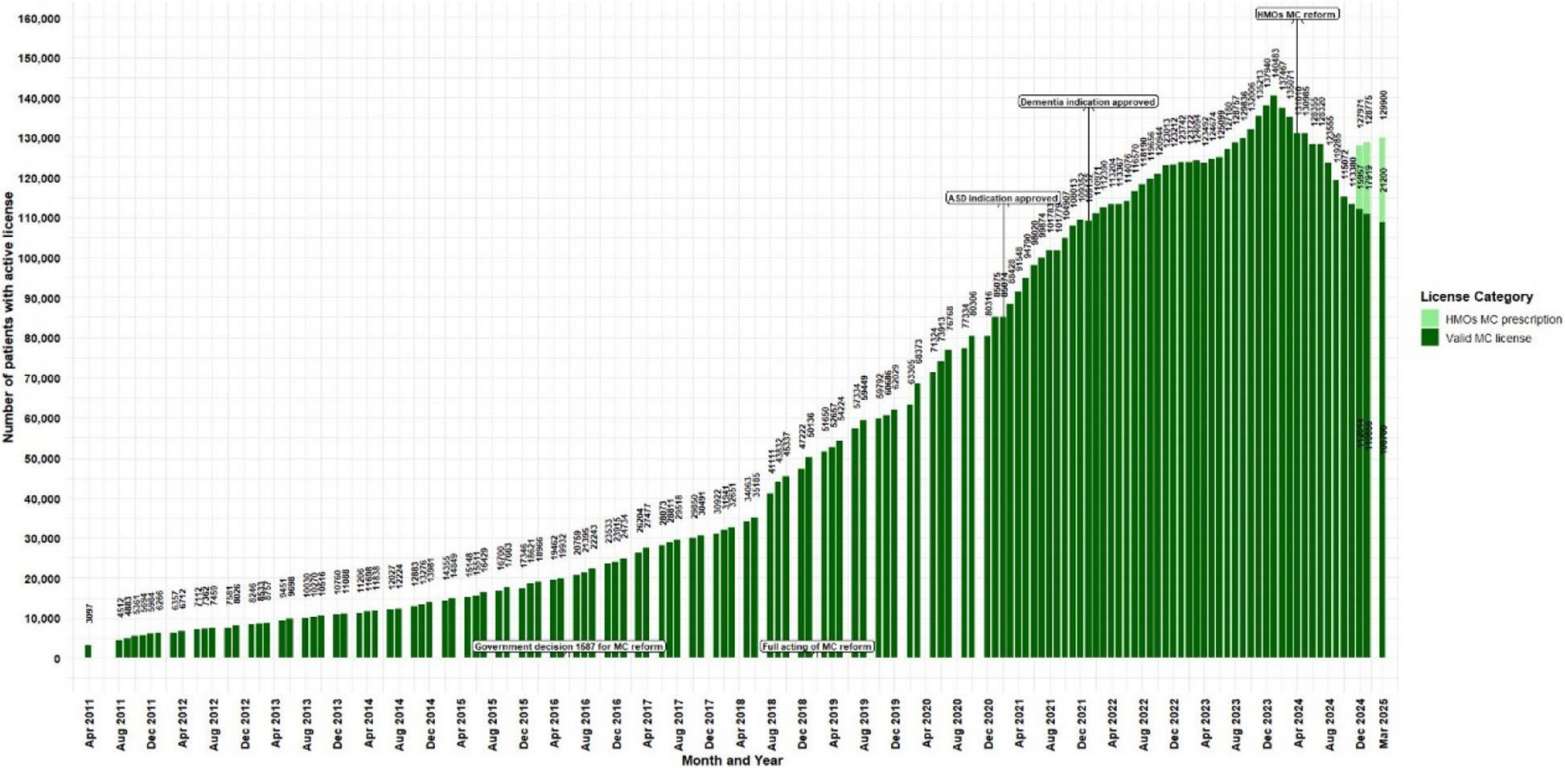
Trends in the number of medical cannabis license holders in Israel from2011 to 2025. Data are presented as monthly counts (N), with significant regulatory eventsindicated at specific months

Botanical cannabis has been regulated and approved in Israel for medical purposes since 1992, under specific conditions (Zolotov et al 2021; The Washingtom Post 2015). Initially, approvals by the Israeli Ministry of Health (IMOH) were granted on a case-by-case basis, primarily for end-of-life patients undergoing palliative care (Schleider et al 2018). Although no official reports provided information on the preliminary period, unofficial reports state that for the first decade, the government issued only 62 patient MC licenses (MAPS 2025). Government resolution #3609 since August 7th 2011, authorized the IMCU oversight over MC treatment (Israeli government 2011). Between those periods (i.e., 1992 to 2011) and until data collection began on April 2011 (i.e., the first data point of the current study), 19 years later, there were 3,097 patients with an active MC license (Fig. 1).

Until December 2013, only IMCU physicians (referred to as "managers") were authorized to approve or renew MC licenses. Physicians who deemed MC appropriate for a patient submitted a digital form to the IMCU, including relevant medical history, indication (from a predefined list), prior treatment attempts, and medication usage. Once submitted (by fax, until around the 2019–2020 when the process transitioned to an online system), it was transferred to an IMCU physician for review, who would decide whether to approve, deny, or request additional information. A similar process was required for license renewal, every 3, 6, or 12 months, depending on several factors. Separate regulations were written for oncology patients, likely due to clinical urgency, wherein 11 oncologists were approved to provide MC licenses directly, without IMCU oversight.

In December 2013, when there were 10,750 active MC licenses, a new directive was issued by the IMOH that expanded authorizing privileges beyond the original 21 IMCU physicians to include 10 additional specialist physicians (specialties not reported) permitted to approve MC licenses for approved indications only within their clinical specialty (Library of congress^2014^); Israeli government 2020), potentially expanding patients access to MC treatment.

At the time of government resolution #1587 dated June 2016, namely, the formal "medicalization" outline of cannabis products (IMOH 2016), there were approximately 19,932 active MC licenses, an increase of 543.5% from April 2011. The next regulatory change was in February 2019, with the full acting of the MC reform that was summarized in the IMCU protocol #106 for Israeli Medical Cannabis – Good Clinical Practice (IMC-GCP) dated July 2016 (3rd version also referred to as the “Green Book”) (Landshaft et al. 2019), with two prior drafts that were not released publicly. In February 2019, there were 50,136 active MC licenses, representing an additional increase of 151.6%.

Important caveat, although not clinical in nature, but which may impact the presented data, is that prior to end of April 2019, out-of-pocket expenses for MC were regulated by the government, for all patients, regardless of their allowed amounts in the active MC license, with a fixed price of 370 New Israeli Shekels (NIS) (~ 100$) and MC was distributed directly by growers to patients (with an optional direct delivery costing extra 100 NIS (~ 27$)). On May 2019 onward, the government transitioned to a market-based per-product pricing model, implemented through pharmacies. It was assumed by the government committee, that for most patients, with monthly allowance quantity of up to 30gr (about half of active licenses were for ≤ 30 g/month at the pricing transition), monthly out-of-pocket costs will be comparable or even reduced. Nonetheless, when this reform was implemented, pharmacies prices ranged between 15–39 NIS/gr, (~ 4–11$), representing 450–1,170 NIS (~ 122–317$) for a 30gr license per month (Israeli government 2019).

Notably, prices steeply increased for MC licenses of 40, 50, 60, 70, 80, 90, 100 and 110, to ranges of 600–1,560 NIS (~ 162–422$), 750–1,950 NIS (~ 203–528$), 900–2,340 NIS (~ 244–634$), 1,050–2,730 NIS (~ 285–739$), 1,200–3,120 NIS (~ 325–846$), 1,350–3,510 NIS (~ 366–951$), 1,500–3,900 NIS (~ 407–1,057$), and 1,650–4,290 NIS (~ 448–1,162$) per month, respectively, depending on the pharmacy and product brand. Notably, although not supported by an official document, these wider, and generally higher costs are likely the byproduct of pharmacy profit margins, stricter GMP standardization requirements, brand dispersion and import dynamics.

On May 2020, about 94 physicians were approved to issue MC license directly to their patients, without IMCU additional approval (Israeli government 2020). A 5th version of protocol #106 was released on January 2021 (Landshaft et al. 2021), at which point there were 80,306 patients, representing a further 60.1% increase. The 7th version of protocol #106 was released on January 2024 (Landshaft et al. 2024), when numbers peaked at 140,483 active MC licenses, with an additional increase of 74.9%, representing a 180.2% increase since the full implementation of the MC reform. From April 2011 (first available data point) to January 2024, a 4,437.2% increase was noted. Under the 2019 reform, per-product pricing and pharmacy channelization replaced the flat-fee license model. Combined with the 2020 decentralization of prescribing authority to trained physicians, these changes aimed to reduce administrative frictions and broaden access, assumably helping to drive subsequent growth in active MC licenses and products variety.

Revisions of protocol #106 primarily addressed additions or removals of approved medical indications in line with ongoing clinical research. The most recent regulatory change was enacted in April 2024 (Israeli government 2024); IMOH 2024), whereby all medical indications, except CNCP and PTSD delegated to the HMOs physicians instead of IMCU-based licensure. From the peak number of active licenses in January 2024, there was a steady reduction to active MC licenses. From December 2024, information was provided on MC prescriptions issued by the HMOs (not retrospectively to the date of the HMOs reform) and by the latest data point published (March 2025), a reduction of 7.5% active MC licenses is observed, reaching 129,900 active MC licenses and prescriptions. Mentionable, similar number that was reported on September 2023. Plausible drivers, while considering the data gaps of the current study may include stricter HMO gatekeeping, non-renewal following irregularities investigations, and anticipatory behavior after reform announcements. Figure 1 displays the main regulatory changes and the discussed trends.

### Gender of patients with active MC licenses

**Fig. 2:**
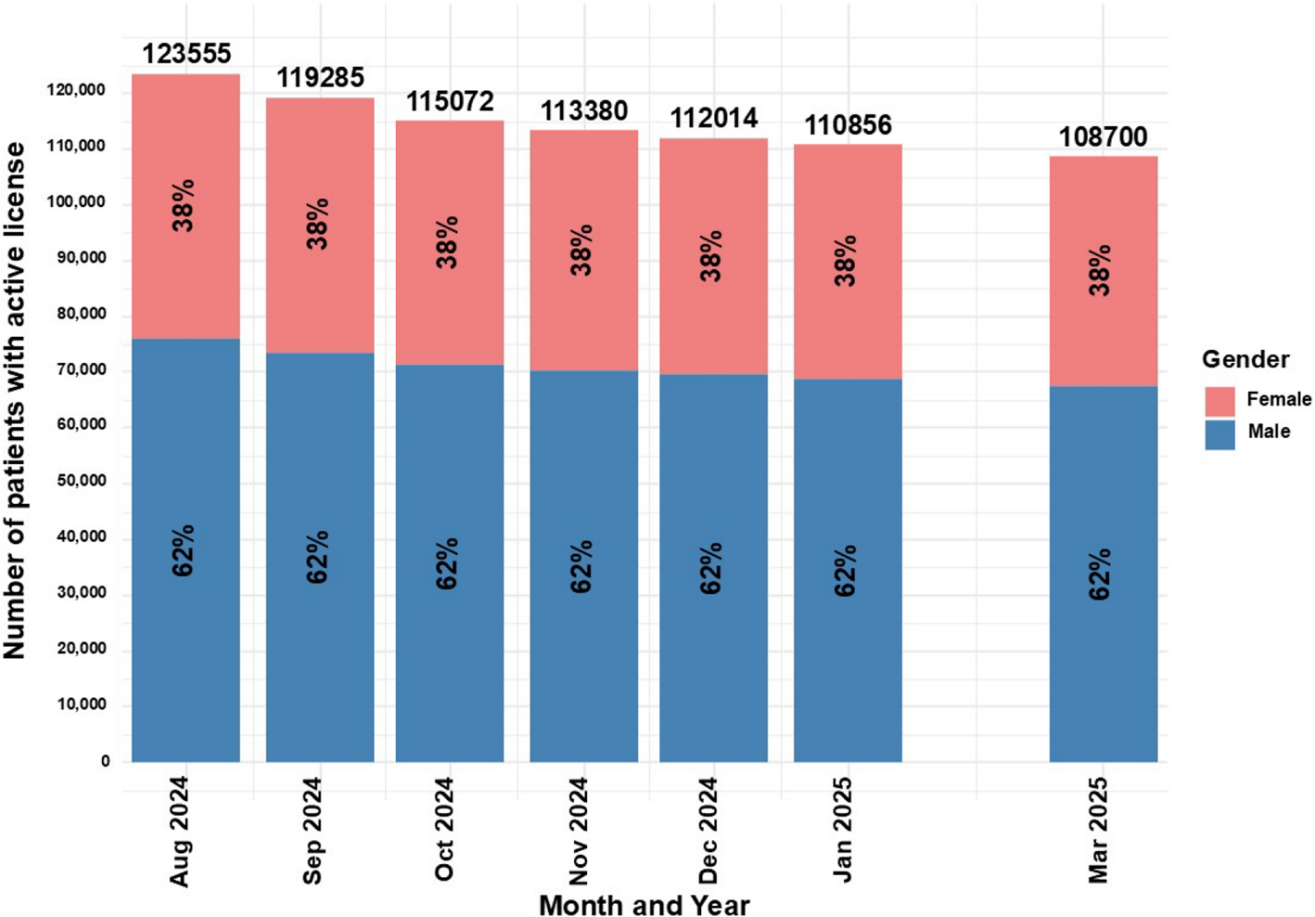
Distribution of gender for medical cannabis license holders in Israel from2024 to 2025. Data are stratified by gender and presented as monthly total counts (N) andpercentage (%) for each gender

For the time points in which gender distribution was reported, regardless of the decrease in the total number of active MC licenses, the proportion between genders remained unchanged, with 62% males and 38% females (Fig. 2).

### Medical indications of active MC licenses

**Fig. 3:**
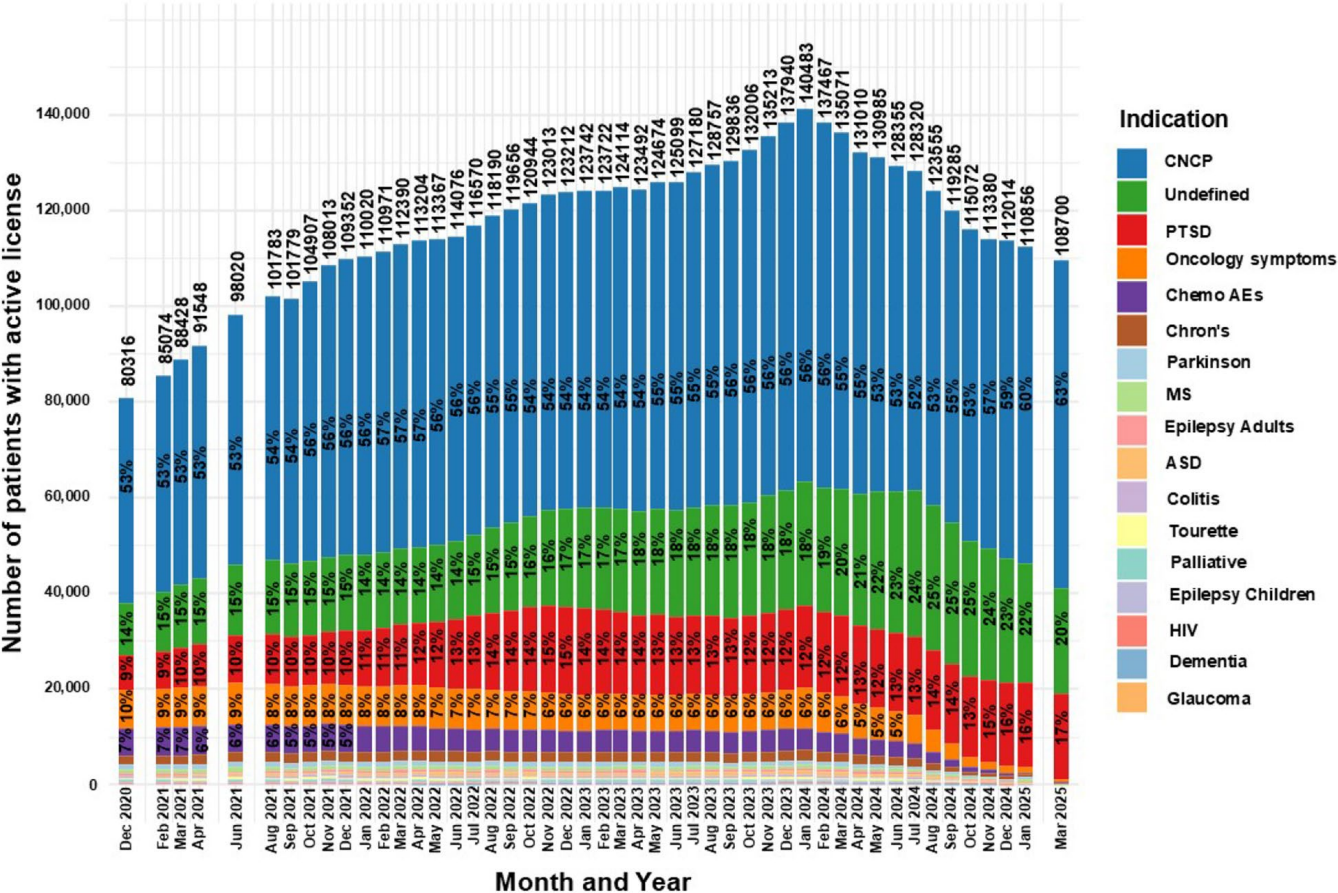
Distribution of licensed indications for medical cannabis license holdersin Israel from 2020 to 2025. Data are stratified by indication and presented as monthly total counts (N) and percentage (%) for each indication

Figure 3 displays proportions of patients’ medical indications for active MC licenses across reported time points. As the distribution of medical indication was majorly affected by the HMOs MC reform in April 2024 (IMOH 2024), and no data was available for indications for HMO prescriptions', this date will be assessed as a milestone in this section.

Some changes can be observed over time, including an increase in the frequency of CNCP, the undefined category and PTSD from 53%, 14% and 9% in December 2020 to 55%, 21% and 13%, respectively, in April 2024, representing a 3.8%, 50% and 44.4% increase. An additional increase was observed in March 2025 to 63%, 20%, and 17%, respectively.

Notably, although the increase in frequencies was linear for CNCP and the undefined category, the number of actual active MC licenses for them fluctuated: from 42,723 and 10,994 in December 2020, increasing to 71,547 and 27,336 in April 2024, and a decrease to 68,398 and 22,203 in March 2025, respectively. The numbers for PTSD increased steadily throughout the period, from 7,329 in December 2020 to 16,478 in April 2024 and 18,074 in March 2025.

Conversely, the frequencies of treatment for oncology symptoms and chemotherapy AEs gradually decreased from 10 and 7% to 5% and 3%, in April 2024, representing a 50% and 57.1% decrease over the same period, and fell further to 0.4% and 0.2%, respectively, in March 2025.

Additional notable observations are gradual decline in frequency of patients with Crohn’s disease, Parkinson’s disease and Tourette syndrome: a trend that began even prior to the initiation of the aforementioned reform. On the other hand, the frequencies of patients with palliative, adult and children epilepsy, colitis and HIV indications were, although low, relatively stable until the enactment of the reform, after which a decrease was observed.

Special cases are ASD, which was approved as an indication for MC in February 2021 and subsequently increased gradually to a peak of 681 patients at the time of the reform. A similar trajectory was observed for dementia, which was approved in January 2022 and reached a peak of 337 license approvals (0.2%), both followed by a decline. Notably, the addition of both indications appears to have had little effect on the overall growth trajectory of active MC licenses leading to their peak in January 2024.

Glaucoma was mentioned in the reports only during the first few months, peaking at 6 patients and later disappearing from subsequent category breakdowns.

### MC monthly amounts

**Fig. 4:**
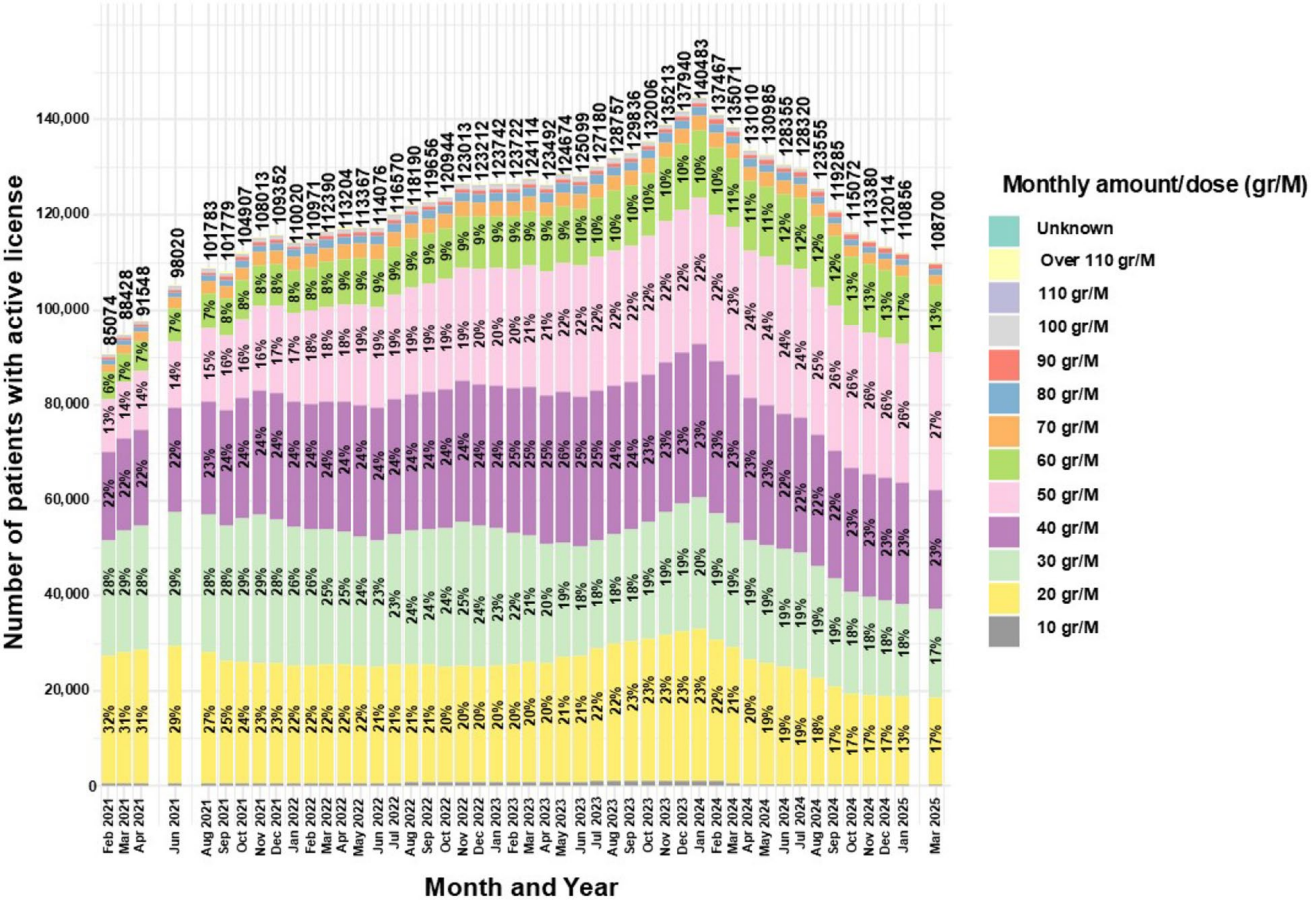
Distribution of monthly MC approved amounts for medical cannabis license holders in Israel from 2021 to 2025. Data are stratified by monthly amount and presented as monthly total counts (N) and percentage (%) for each monthly amount

Figure 4 summarizes shifts in approved monthly allowance (gr/month) between February 2021 and March 2025. The 20gr and 30gr categories fell from 32 and 28% to 17% and 17% (−46.9% and −39.3%), while 50gr and 60gr rose from 13 and 6% to 27% and 13% (+ 107.7% and + 116.7%). The 40gr category remained the modal band, consistently ~ 22–25% (23% in March 2025). Less common bands changed modestly: 70gr was stable (~ 2.05% to 1.94%), 80gr fluctuated slightly (~ 1.09% to 1.18%), 90gr increased (0.52% to 0.95%), and ≥ 100gr bands declined (100gr 0.60% to 0.14%; 110gr 0.04% to 0.01%; > 110gr 0.39% to 0.04%). Licenses for 10 gr/month remained rare (< 0.5%). Because dose-band data begin in February 2021, potential effects of the May 2019 pricing reform (Israeli government 2019) cannot be assessed.

### HMOs of patients with active MC licenses

Given the potential influence of the April 2024 HMOs MC reform (IMOH 2024), trends are assessed in that context.

**Fig. 5:**
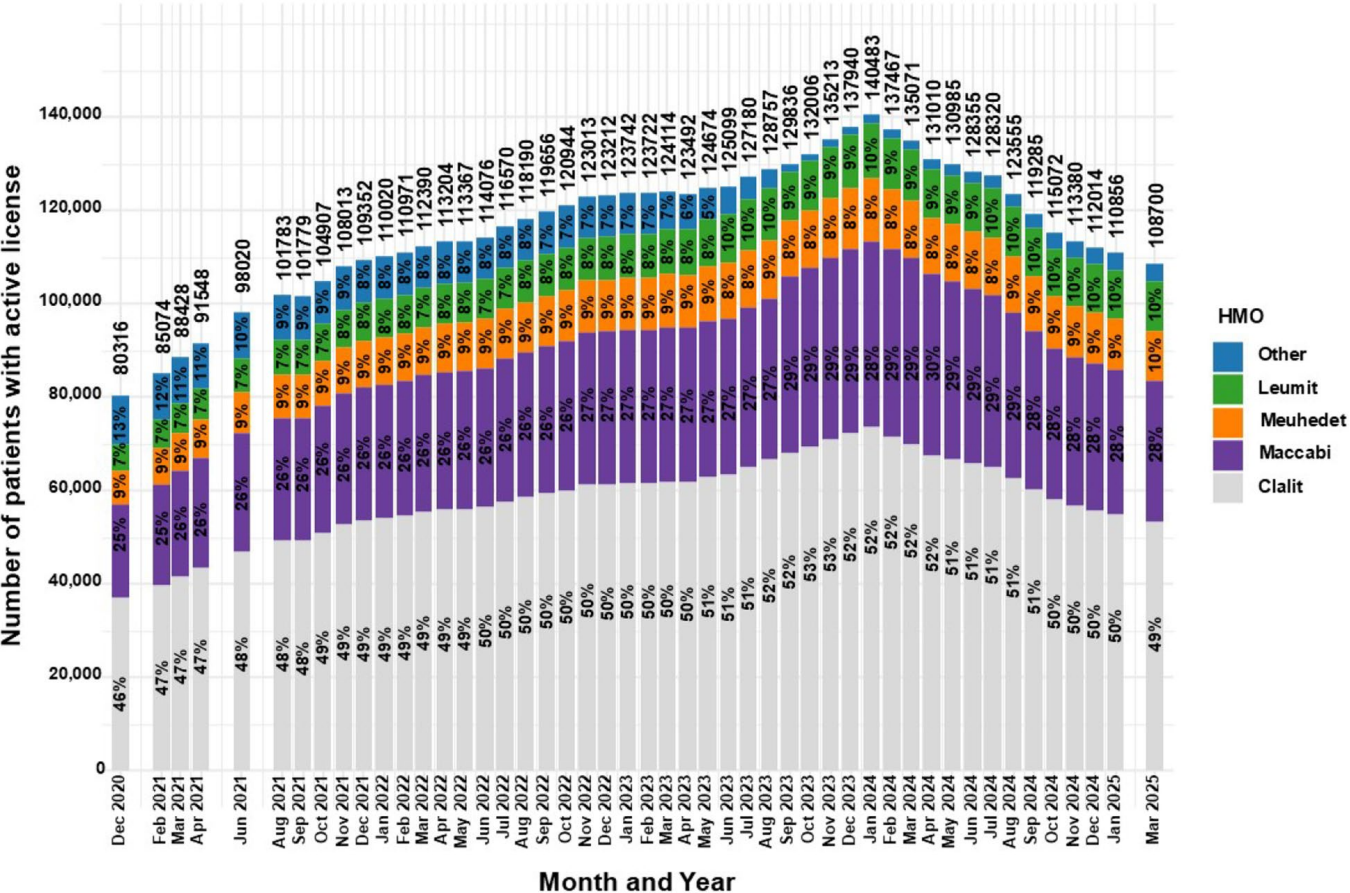
Distribution of HMOs for medical cannabis license holders in Israel from 2020 to 2025. Data are stratified by HMO and presented as monthly total counts (N) and percentage (%) for each HMO

As observed in Fig. 5, Between December 2020 and April 2024, frequencies of patients with active MC licenses insured by Clalit, Maccabi, and Leumit increased from 46%, 25%, and 7% to 52%, 30%, and 9%, respectively, representing relative increases of 13.0%, 20.0%, and 28.6%.

By March 2025, Leumit rose further to 10%, while Clalit and Maccabi declined to 49% and 28%, respectively.

Meanwhile, the “Other” category declined from 13% in December 2020 to 1.73% in April 2024, but rebounded to 3.65% by March 2025, representing a 111.0% relative increase.

### Cumulative MC allowance based on administration routes of active MC licenses

**Fig. 6:**
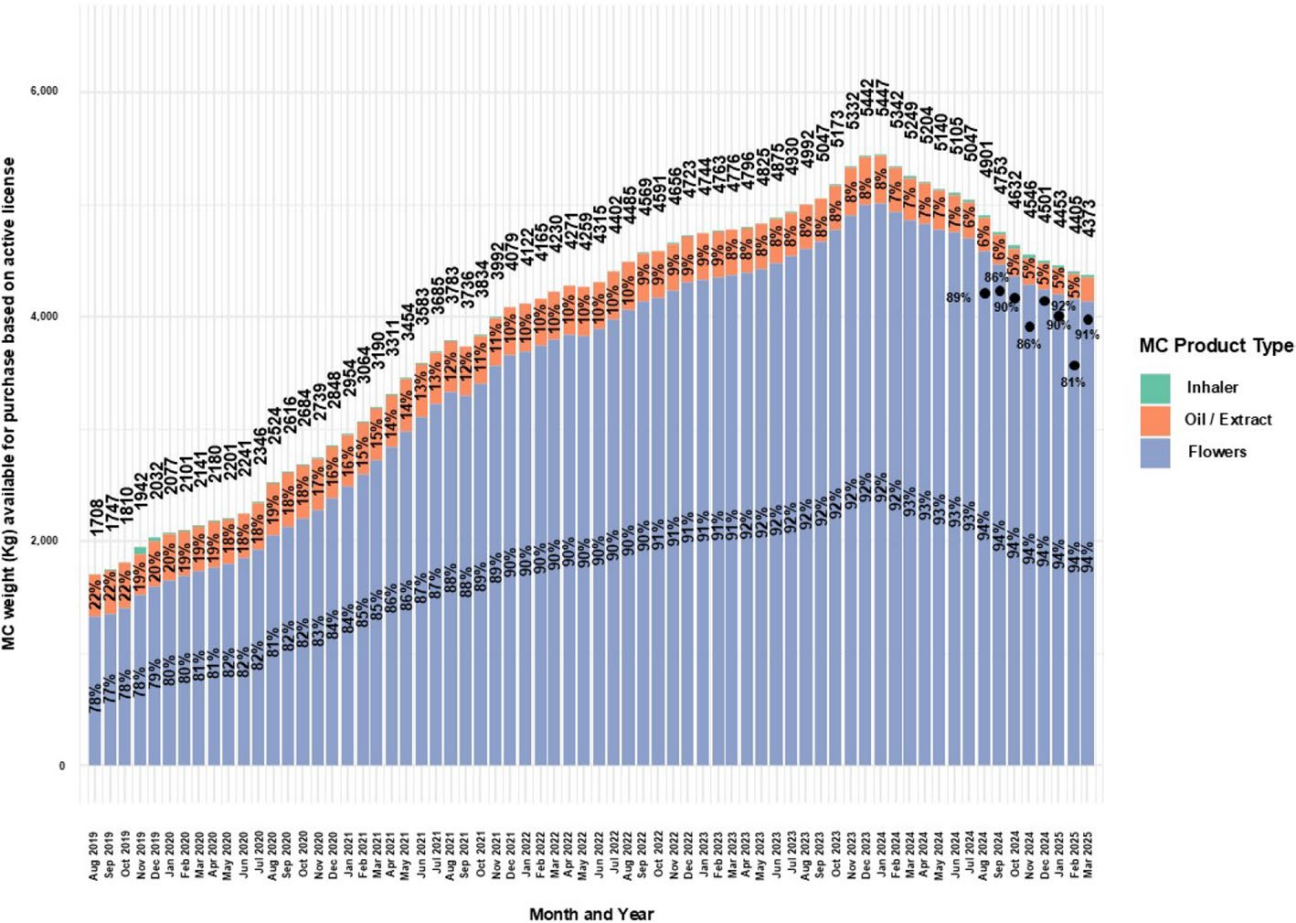
Distribution of approved monthly weights of MC for medical cannabis license holders in Israel from 2019 to 2025. Data are stratified by administration route and presented as monthly total counts (N) and percentage (%) for each administration route

Figure 6 displays MC administration routes and approved cumulative allowance.

In parallel with the growth in active MC licenses (Fig. 1), the approved monthly allowance rose from 1,708 kg in August 2019 to a peak of 5,447 kg in January 2024, a 218.9% increase, followed by a decline to 4,373 kg in March 2025, a 19.7% decrease.

Throughout the period, flowers remained the most frequently approved MC product.

Approval for flower MC products increased from 78% in August 2019 to 94% in March 2025 (20.5% increase), while oil MC products dropped from 22% to 4.9% (77.7% decrease).

When available, data showed that patients purchased between 81%–92% of their approved monthly MC allotments.

For inhaler MC cartridges, approvals rose from 0% in August 2019 to 3% in October 2019, then declined steadily to 0.1% and rose again to 0.5% by March 2025—a 400% increase from the recent low.

**Fig. 7:**
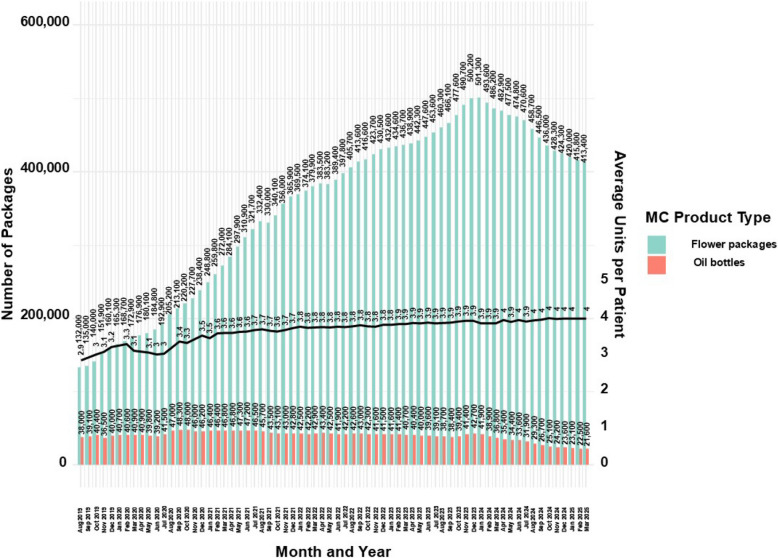
Distribution of approved monthly MC main administration routes number of packages for medical cannabis license holders in Israel from 2019 to 2025. Data are stratified by main administration routes and presented as monthly counts (N) per administration route with calculation results of average monthly products approved per patient

Figure 7 provides an additional view of MC product availability, specifically for the most common types, flower and oil, which reflect the trends seen in Fig. 6.

While flower products increased in proportional frequency through March 2025 (Fig. 6), the absolute number of approved flower packages rose from 132,000 in August 2019 to a peak of 501,300 in January 2024 (279.0% increase), followed by a decline to 413,400 in March 2025 (17.6% decrease).

Thus, despite the growing share of flower products, the declining number of active MC licenses (Fig. 1) limited overall sales volume.

Approved oil bottles rose from 38,000 in August 2019 to 42,700 in January 2024 (12.4% increase), then dropped to 21,600 by March 2025 (49.4% decrease), aligning with trends in active MC licenses (Fig. 1) and oil product proportions (Fig. 6). Combining flower and oil products, the average monthly units per patient rose from 2.9 in August 2019 to 4.0 in March 2025 (37.9% increase), corresponding to an average approved dose of 40gr per month per patient.

### MC import to Israel

**Fig. 8:**
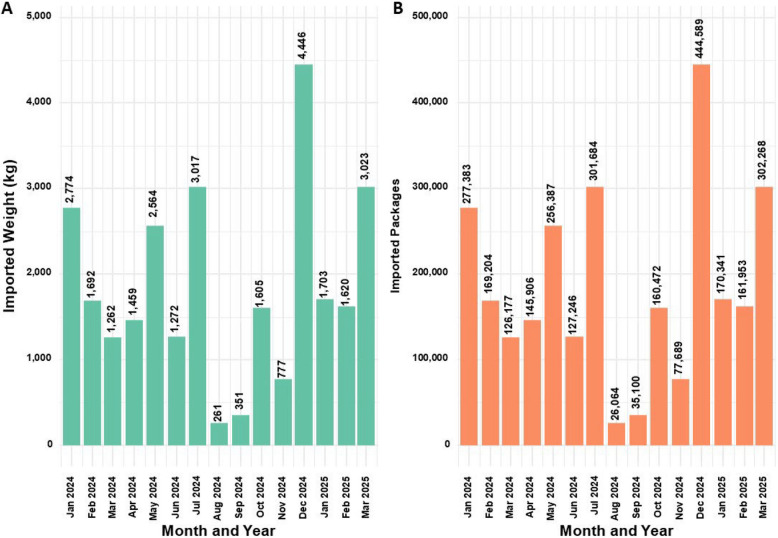
Trends in import volumes (kg) and packages numbers of medical cannabis flower products from 2014 to 2025

Figure 8 displays the trends in imported MC flower quantities (Kg) (A) and package counts (B) to Israel during the reported period.

Substantial fluctuations in import volumes are evident, and these do not always correspond with other trend patterns discussed in this study.

Of note, the highest recorded import occurred in December 2024, just one month before the peak in active MC licenses (Fig. 1), with 4,446 kg (equivalent to 444,589 flower packages) imported. This volume constituted the equivalent of approximately 88.7% of the domestic availability at that time (501,300 flower packages; see Fig. 7).

## Discussion

This study aims to present the full chronicles of MC in Israel, from its inception to current times. The current study results showed consistency with prior publication of MC licensure trends for 2013–2018 (Sznitman 2020), but with added value of more granular (monthly rather than annually) data, an extended study period, and additional measurements that were not available previously. Furthermore, the current study displays the changes in MC regulations alongside observed MC licenses trends, to inform future MC regulators and associated parties about the consequences of government decisions on patients.

In practice, the most important metric for MC patients, physicians, regulators and the industry is the number of monthly active MC licenses. This figure allows patients to estimate the likelihood of continuity in their treatment, while physicians treating patients with MC can anticipate workloads related to license or prescription initiation and renewals. In turn, regulators may assess the effects of policy changes, and the industry can plan accordingly. For example, MC growers might forecast future market demands and determine optimal planting strategies to meet patient needs while minimizing resource waste. However, due to the incompleteness of the publicly available data presented in this study, it is currently speculative as many factors that cannot be controlled may affect the future status of the Israeli MC market.

The current study shows that up until a few months prior to the full enactment of the MC reform (Landshaft et al 2019), there was a slow and steady increase in the number of active MC licenses. This trend (~ 151% increase) is similar to that previously reported for the 2013–2018 period (~ 121% increase) (Sznitman 2020). The inflection point was likely one of the reforms, as following its implementation and the publication of protocol #106 (Landshaft et al 2019), the number of active MC licenses increased sharply, despite intermittent stagnation, peaking in January 2024.

From that point onward, a consistent and substantial decline in active MC licenses was observed through March 2025. This reduction is presumably linked to the HMOs prescription reform enacted in April 2024 (Israeli government 2024), which transferred prescription authority for most indications from the IMCU to the HMOs. However, the decline began even before the reform’s official implementation. This may have been triggered by early media reports announcing the proposed reform as early as December 2023 (Cohen 2023; IMOH 2023) and even initial hints in August 2022 (IMOH 2022). While these regulatory changes should have resulted in license replacement and perhaps even an increase by HMO-issued prescriptions, and some transition lag is expected in large-scale bureaucratic processes, the combined dataset from both IMCU and HMO sources, nearly a year post-reform, still shows a noticeable halt in the number of active MC patients.

Another possible explanation for the observed decline and stagnation is the exposure of licensing irregularities during the previous growth period. Reports have suggested that many licenses may have been issued under questionable circumstances (Levi L 2022). In particular, several physicians had their IMCU authorizations revoked after allegedly issuing licenses from private clinics rather than within public healthcare settings, as required. In one prominent case, a single physician was responsible for over 13,000 license approvals (Cannabis magazine (Cannabis 2022)). Consequently, once those licenses expired, many were likely not renewed by other physicians, contributing to the observed decline. Internationally, access models vary: in the UK, most MC products are prescribed in independent (private) clinics under Care Quality Commission (CQC) oversight with only narrow NHS pathways (NICE 2021; NHS 2019; QCQ 2025). Similarly, US federal settings (e.g., Veteran Affairs) clinicians cannot prescribe or certify (US Department of Veterans Affairs 2023), effectively shifting access to private clinics under state programs. Whereas Italy embed prescribing and dispensing in public health systems (Chambers and Partners 2025).

The gender distribution of patients holding an active MC license remained consistently male-dominant (62%) in the current study. This finding is similar to previous reports (68.5%) (Sznitman 2020), although the gender data for this study was available only for 2024–2025, whereas the prior report covered 2013–2018. One explanation for this finding may be the higher rate of MC-related adverse effects among females, as was found in a previous study published by the author of this study (Aviram et al. 2021), which may discouraged women from pursuing or renewing their MC license. Israeli clinical guidance for oil extract and CBD-rich products preference, treatment initiation with lower THC potency, cautious maximum dose and slower titration (i.e., "start-low, go-slow") was published for pain physicians in 2023 (Brill et al 2023). Nonetheless, subsequent years females' lower frequency does not seem to be effective, possibly as this guidance is not gender specific. Thus, there is a need for further sex-stratified safety/effectiveness research. Another reason may be the relatively high proportion of active MC licenses for the indication of PTSD, which in Israel is predominantly combat-related, thus, predominantly male (Solomon 1989). Nonetheless, the most frequent MC indication was found to be CNCP, which in a recent epidemiological survey in Israel, is more frequently female dominated (~ 60%) (Sharon et al. 2022). However, in a study of over 1,000 eligible MC patients for CNCP in Israel from our lab, the sample was predominantly male (~ 57%) (Aviram et al. 2020b). An additional possible explanation is that female-predominant CNCP diagnoses, such as fibromyalgia and endometriosis, are not approved by the IMCU for MC license (Landshaft et al 2024), which may result in rejected applications or prolonged approval timelines (Aviram et al. 2020b). Compared with the United States (US) MC programs, in a recent analysis of publicly available data from seven states, male-to-female ratio was 102.1 (~ 49.8%) (Boehnke et al. 2025). This difference supports the current study hypothesis about the uniqueness of the Israeli MC patient's population. Unfortunately, there is no additional comparisons to be made as the US programs reported on different measures than the Israeli IMCU did, such as continuous age, percentage of minorities and ethnic groups, household income, unemployment, poverty, disability and veteran rates.

During the investigated period, although there were fluctuations in total numbers of active MC licenses and changes in regulatory framework, CNCP, PTSD and the undefined category proportions increased substantially. In contrast, most of the other medical indication proportions decreased substantially up to the time the HMOs MC prescription reform was enacted on April 2024 (Israeli government 2024), and following this reform, almost disappeared, presumably as all medical indications other than CNCP, PTSD and the undefined, were transferred to the authority of the HMOs. The previous trends report demonstrated a ~ 28% and ~ 1044% increase in active MC licenses for CNCP and PTSD, respectively, between 2013–2018 to frequencies of ~ 59% and ~ 10%, in 2018, respectively. Additionally, the previous report showed a ~ 69% decrease in the undefined category to ~ 12% in 2018 (Sznitman 2020). As the current report data on these measurements begin in December 2020, those trends cannot be compared directly, but may serve as an earlier extension and validation of the current report initial data point. As such, CNCP, PTSD and the undefined frequencies in December 2020 were 53%, 14%, and 9%, respectively. Considering the two years gap of information between the reports on this measurement, a ~ 10% and ~ 25% decrease was observed for CNCP and the undefined category, respectively, and a ~ 40% increase for PTSD. The small decrease in CNCP proportions during this gap might be explained by the changes in data structure or emerging clinical guidelines at that time, which advised CNCP specialist physicians to assert caution before prescribing a cannabis-based medicine due to lack of high-quality controlled trials, and to do so only as a third-line treatment and only for chronic neuropathic pain (Häuser et al. 2018). The additional decline in the undefined category proportions may be explained by improved physician education and more accurate form completion (IMOH 2023), or by the full enactment of the MC reform which clarified approved medical indications (Landshaft et al. 2019).

Notably, between 2020 and 2025, the proportion of the undefined category increased, contradicting expectations of continued decline from the previous report (Sznitman 2020). This may be explained by the rapid expansion in active MC license numbers, which may have led to increased data entry inconsistencies due to overload on physicians and IMCU processes. PTSD proportions extended between reports and across the entire current study period, being the only indication that increased both in proportions and sample size, even following the total decline in MC licenses and the HMOs prescription reform. Unfortunately, this is easy to explain. As the only Jewish country in the Middle East, Israel has been subjected to many wars since its inception, and many of its citizens are exposed to continuous traumatic stress, leading to PTSD and depression (Green et al. 2017). A recent projection estimated that 5.3% of the population (> 520,000; CI: 1.64–9%) may develop PTSD due to the ongoing war (Katsoty et al. (Katsoty 2024)). Perhaps the most convincing explanation is economic: As PTSD in Israel is mainly combat-related, many patients are treated under the Department of Rehabilitation of the Ministry of Defense, receiving full reimbursement for MC costs (Israeli department of rehabilitation 2025). Importantly, this increase is not in accordance with clinical guidelines, which strongly recommend against the use of MC for PTSD due to a lack of high-quality studies (Lang et al. 2024). Still, the growing number of PTSD patients receiving MC may have led psychiatrists to gain clinical experience supporting its perceived benefits. Additionally, regulations for MC license approval for PTSD remain stricter and require longer period of conventional treatment exhaustion attempts than for other approved indications (Landshaft et al 2019).

The small observed increase in MC licenses for ASD and dementia is likely driven by recent clinical studies demonstrating potential therapeutic benefits (Hermush et al. 2022; Hacohen et al. 2022; Schnapp et al 2022).

The current study provides a novel analysis of monthly MC amounts permitted under active MC licenses, as this variable was not reported in previous literature. Data were available for the years 2021–2025. According to the MC regulations in this period, the initially approved monthly amount could not exceed 20 gr (Landshaft et al 2019), and therefore, almost all patients were provided with this amount at first (Aviram et al. 2020b). The decrease in the proportion of this initial amount may reflect a lower proportion of newly issued MC licenses compared to renewals. A similar trend of reduction in proportions was found for 30 gr/M. Based on the regulations, for any change in the license, including dose increase or product type change, specific documents must be submitted by the treating physician for approval by the IMCU (Landshaft et al 2019). The reduction in the 30 gr/M category may follow the same logic, as it represents the first approved dose increase.

Trends are different for the category of 40 gr/M, which remained relatively stable between 22%–25% throughout the study period. This stability may be attributed to most patients not requiring more than this monthly amount. It might be explained by the dosing guidelines for MC (Bhaskar et al 2021), calling for a maximum daily dose of 40 mg of ∆−9-tetrahydrocannabinol (THC) by oral oil/extract formulations. Given that many Israeli patients use THC-rich MC (~ 20% potency, or ~ 200 mg/gr), particularly those with CNCP (Bar-Lev Schleider et al. 2022), and that most choose flower products (Aviram et al. 2020b), the theoretical requirement to reach the recommended 1,200 mg/month THC dose would be ~ 6 gr/month of MC. However, considering the significant blood concentration variability (2%–56%), dependent on inhalation technique, device design, lung capacity, and user behavior, and with ~ 30% of THC assumed to be destroyed by pyrolysis (Lucas et al. 2018; Grotenhermen 2003; Huestis 2007), patients may require up to 40 gr/M of raw MC flowers.

Nonetheless, the current study shows a trend for substantial increase in the proportions of 50 gr/M and 60 gr/M categories. This finding is harder to explain given the aforementioned guidelines for MC dosing. Some evidence on CNCP patients, shows that clinical practice may allow for a limit of 60 gr/M, when clinically justified (Robinson et al. 2022). Higher monthly amounts of 70–110 gr/M or more are infrequent and showed a decreasing trend, almost disappearing. This trend may be attributed to an IMCU initiative to reduce licenses exceeding 90 gr/M (Cannabis magazine 2019). Another reason for these relatively high amounts might be their use in producing Rick Simpson Oil (RSO), requiring up to 180 gr for production. Although RSO is largely unstudied clinically, it is perceived by some as an anticancer treatment (Guggisberg et al. 2022). Notably, there may be a risk of secondary gain by diversion of MC to the general public, a risk that may increase with higher monthly allowances. A recent study showed that despite being informed that diversion on their part constitutes drug trafficking, most patients still perceived it as at least moderately moral (Ne’eman-Haviv and Rozmann 2023).

Another novel variable analyzed in this study is the trend in active MC licenses by HMOs affiliation, which has not been previously reported. Although MC expenses are not reimbursed by the HMOs in the majority of cases due to its regulatory definition, since the enactment of the National Health Insurance Law in 1995, all Israeli permanent residents are automatically covered by the public health system (Israeli government 1994). Therefore, every patient holding an MC license is affiliated with one of the four national HMOs.

This information became more important due to the HMOs prescription reform, which delegated prescribing authority for all indications except CNCP and PTSD to HMO-affiliated physicians, starting in April 2024 (Israeli government 2024). A decline began after the reform’s announcement (December 2023) and continued through implementation (April 2024), consistent with both anticipatory behavior and subsequent stricter prescribing as well as purge of irregular licenses and transition frictions. If regulatory control of CNCP and PTSD is also eventually transferred to the HMOs, the data presented here may assist in workload forecasting and resource planning, although the IMCU and the HMOs have not reported that this is in their scope at the time this study was submitted.

This corresponds with Clalit’s position as the largest HMO in Israel, insuring 52% of the population (Sagy et al 2025). In general, the proportion of active MC licenses affiliated with Clalit and Maccabi increased until shortly before the HMOs prescription reform, after which a modest decline was observed. Shifts in HMO shares may reflect member churn, coverage/routing differences and regional mix. Specifically, a reduction in the "Other" category, which likely captured cases with missing HMO information, since no additional HMOs exist in Israel. Conversely, the observed decline in Clalit and Maccabi proportions may be due to a relative increase in Meuhedet and Leumit affiliations during the same period.

Another novel aspect of this study is the analysis of MC product type trends, along with the estimation of monthly flower package and oil bottle volumes based on active MC licenses, and the average number of product units available per patient. The current study results demonstrate that the most prevalent MC product type is MC flowers, which increased over time to reach 94% of all dispensed product allowance. In contrast, the proportion of MC oil products declined to under 5% of the total allowance. These results correspond with what was reported (86%) among CNCP patients during 2018–2020 prospective one-year study, and also show a similar trend of increasing flower use (mostly via smoking) and decreasing oil purchases (Aviram et al. 2020b).

This contradicts medical guidelines, which generally discourage smoking (Häuser et al. 2018). A possible explanation is the higher short-term adverse event rate associated with MC oil (~ 52%) (Pud et al. 2024), compared with smoking (~ 40%) (Aviram et al. 2020b), which may discourage its use.

Inhaler frequency steadily increased to 0.5%. Notably, unlike MC flowers and oil products, the inhaler utilizes a much lower amount of MC flowers, specifically 13.5 ± 0.9 mg per inhalation and 0.81 g per 60-inhalation cartridge (Aviram et al. 2022). Thus, while the inhaler accounts for just 0.5% of total product allowance (in Kgs), it may represent a disproportionately large share of patients. This pattern aligns with broader observations that inhalation remains common among MC patients in a recent European survey (Fortin et al. 2025).

Although data on actual patient purchases are available only for recent months, these figures may shed light on potential discrepancies between licensed and consumed quantities. This gap could suggest overprescription (i.e., allocation of unnecessary monthly amounts), or it may reflect economic barriers, as market prices (Israeli government 2019) ranging from $122 to $1,162 per month depending on the approved amount. This price dispersion likely reflects pharmacy margins, GMP standardization costs, product brand segmentation, and import dynamics under the market-based model. Another possibility is that some patients discontinue treatment but retain an active license without making any purchases.

Allowance data from the IMCU on active MC licenses also enables estimation of market share for MC growers and companies. At its peak in January 2024, the total monthly allowance reached 501,300 flower packages and 41,900 oil bottles. Assuming an average product price of $40–$110 (Israeli government 2019), this equates to a potential market value of approximately $21.7–59.5 million per month (annualized $252–$684 million, under the same assumptions). However, these figures apply only to IMCU-issued licenses and not to HMOs-issued prescriptions, and therefore may not fully reflect post-April 2024 market dynamics (Israeli government 2024). Separately reported import data show that, in December 2024, just before the MC license peak, the estimated value of imported flower products ranged from 17.7$ to 48.9$ million. Given the capped total MC supply, imports likely displaced a portion of the domestic market. Imports were initially approved by the Israeli government in response to rapid demand growth that caused product shortages (Pilot A 2019). By early 2024, however, domestic producers began petitioning the government to address market saturation caused by excessive imports, particularly from Canada (Israeli government 2024). Despite this, 302,268 imported MC flower packages were reported as recently as March 2025.

Throughout the study period, the average number of MC product units (flower packages and oil bottles) per patient steadily increased, reaching 4 units per patient by March 2025. This finding aligns with previous research from our lab indicating that the majority (51%) of male MC patients in Israel, licensed for durations ranging from 1 to 13 years, consumed between 40 and 100 g per month, with a median of 40 gr/M (Aviram et al. 2021).

### Limitations

This study is subject to several important limitations. First, monthly reports from the IMCU were not publicly available for the full regulatory period (1992–2025). Specifically, no official data was found prior to April 2011, when systematic data collection began, and reports with adequate data beyond March 2025 were not yet released at the time of submission. Additionally, many measurements were inconsistently reported across time, with key variables, such as product type, dose distribution, and patient demographic breakdowns, being introduced or omitted across various months, limiting continuity of analysis. In several cases, values reported for earlier months were retrospectively updated in subsequent reports, introducing possible biases or discrepancies in longitudinal trend assessments. A formal response from the IMCU responded that the reports are based on accurate data from a computerized system, and although some deviation is to be expected in a retrospective report, those are minimal and would not affect any trend analysis.

Another substantial limitation concerns the April 2024 regulatory reform (Israeli government 2024), which shifted prescription authority for most MC indications from the IMCU to HMOs. Although this policy change represents a major structural shift, data on HMO-issued prescriptions was not made available between April and November 2024. Even after data publication resumed in December 2024, reporting remained non-granular, lacking stratification by demographics, dosage, or clinical indication for the HMOs prescriptions data. Furthermore, the data do not distinguish between prescriptions still under IMCU control (i.e., CNCP and PTSD) and those issued directly by HMO physicians, making it difficult to interpret post-reform dynamics for specific patient groups.

Another important limitation is the absence of patient-level data. This analysis relied exclusively on aggregate population-level statistics provided by the IMCU and MOH, precluding examination of individual treatment courses, retention rates, or clinical outcomes. Notably, a formal response from the IMCU responded that they could not share individualized, anonymized patients' data due to medical confidentiality. Moreover, no publicly available IMCU reports included data on product safety, adverse events, or quality control parameters, factors essential to assessing long-term treatment implications.

The discontinuity in reporting formats and shifting definitions across time periods further limits data comparability. For instance, the “undefined” indication category emerged only after 2020, raising the possibility that some changes reflect reporting artifacts rather than genuine clinical trends. Additionally, the study may be subject to selection bias, as the analysis includes only patients who successfully navigated the licensing process, potentially underrepresenting underserved populations or those unable to access authorizing physicians, especially in earlier years of centralized approvals.

Lastly, the transition in May 2019 to a market-based pricing model introduced variability in out-of-pocket costs. Although assumed to reduce financial burden for many patients, the absence of socioeconomic data or insurance status in the IMCU reports limits the ability to evaluate the reform's equity implications or its potential influence on prescribing patterns and treatment access. In addition, without any control for external market value factors which might have affected the calculated market value estimates, these economic calculations remain limited and should be interpreted carefully. Nonetheless, additional analyses with alternative data sources such as pharmacy sales could have enhance the research robustness but those cannot be made as this data is not available to the public.

In an attempt to address these gaps, the author contacted relevant IMCU representatives via formal written correspondence between June 12 to September 7, 2025. During the revision, a response was received, and although some information has been provided and integrated into this paper analyses', access to the full dataset remains limited, and several important questions remained unanswered. These limitations should be considered when interpreting the trends presented in this study.

## Conclusions

This study provides the most comprehensive analysis to date of Israel's MC program, offering a longitudinal, granular view of licensure trends, regulatory impacts, patient demographics, medical indications, dosing patterns, product types, and market dynamics from 2011 through early 2025. By integrating monthly data with regulatory milestones, this work offers critical insights into how policy shifts directly influence clinical access, patient behavior, and industry operations. The observed post-HMO-prescription-reform decline in active MC licenses, the shift in patient demographics and medical indications, and the evolving product preferences highlight both the sensitivity of the system to regulatory design and the importance of evidence-based policymaking. As Israel continues to serve as a global model for national MC programs, future reforms should be informed by real-world data trends such as those presented here, ensuring patient continuity of care while preserving regulatory integrity and system sustainability.

Future studies should examine the clinical and economic impacts of the 2024 regulatory reform, with particular focus on prescription trends, patient outcomes, and equity in access across HMOs. Additional research is needed to assess the long-term effectiveness and safety of different MC product types, and to explore real-world prescribing practices versus guideline recommendations. Integration of granular HMO prescription data will be essential for monitoring treatment patterns, understanding potential underuse or diversion, and supporting evidence-based policy development.

## Data Availability

All data analyzed during this study are included in this published article and are publicly available through the monthly reports released by the IMCU. Supplementary materials including the processed datasets are available from the corresponding author upon reasonable request.

## Abbreviations

ASD: Autism spectrum disorder
CBD: Cannabidiol
CNCP: Chronic non-cancer pain
Gr/M: Grams per Month
GCP: Good clinical practice
HMO: Health maintenance organization
IMCU: Israeli medical cannabis unit
IMOH: Israeli ministry of health
MC: Medical cannabis
Mg: Milligram
NIS: New Israeli Shekel
PTSD: Post-traumatic stress disorder
RSO: Rick Simpson oil
RCT: Randomized controlled trial
THC: Δ9-tetrahydrocannabinol
US: United States
